# Supplementary figures and images for: Improvement of enzymatic saccharification yield in Arabidopsis thaliana by ectopic expression of the rice SUB1A-1 transcription factor
Source: PeerJ. 2015 Mar 3;3:e817. doi: 10.7717/peerj.817 (PMC4358655; doi:10.7717/peerj.817)

mg of glucose g<sup>-1</sup> FW

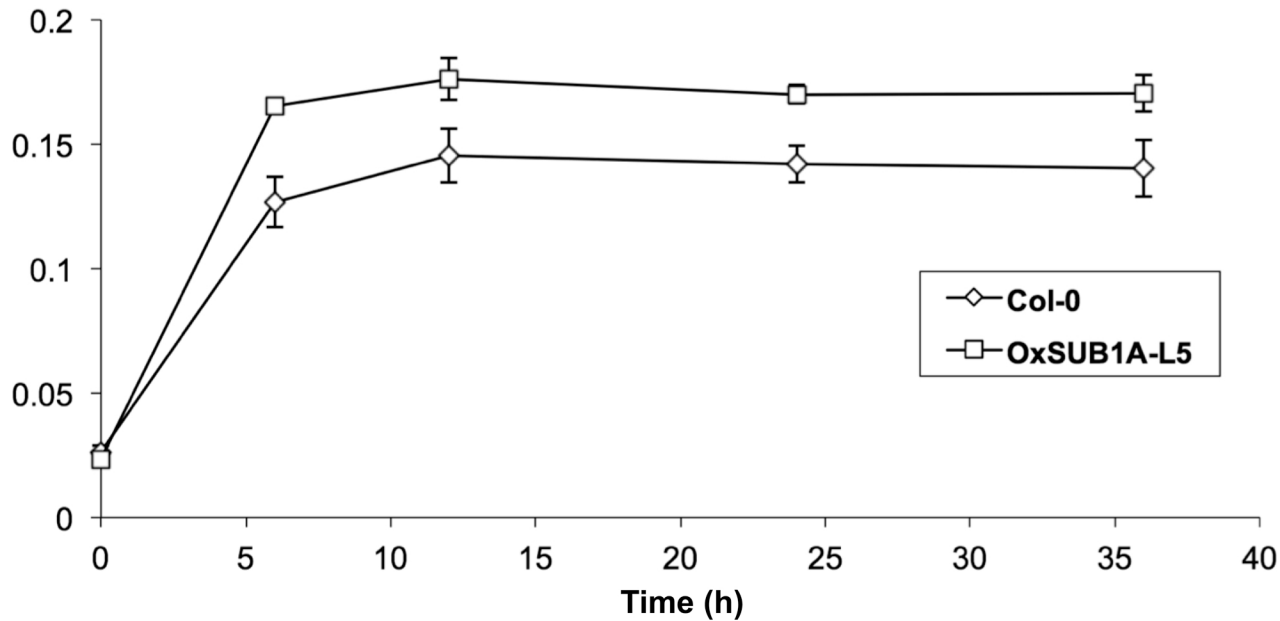

Supplement: Figure S1 — Glucose content was measured with glucose oxidase. 22-day-old plants. Values are means of two independent experimental replicates, each with n = 3 plants. Error bars are ±S.E. [file peerj-03-817-s001.pdf]

**Wild-Type  
(Col-0)**

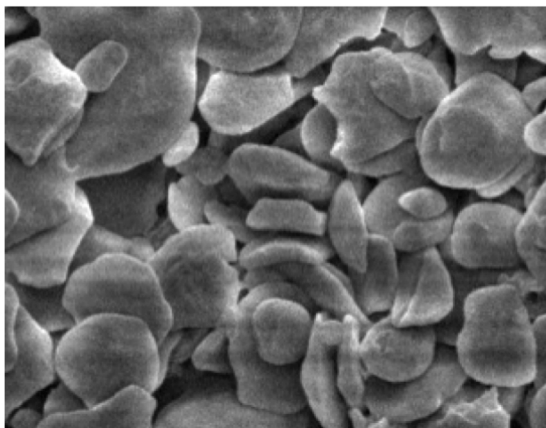

**OxSUB1A-L5**

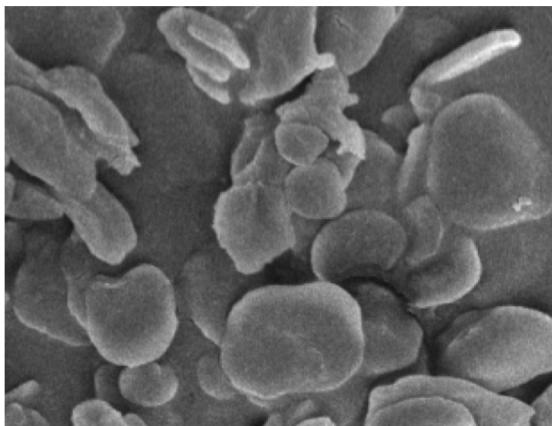

**OxSUB1A-L12**

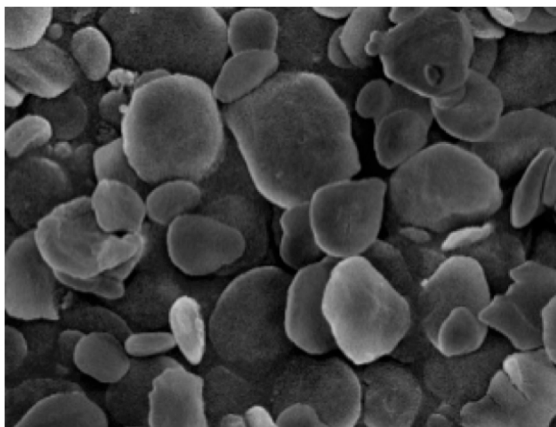

Supplement: Figure S2 — Black bar is 10 µm. [file peerj-03-817-s002.pdf]

**(A)**

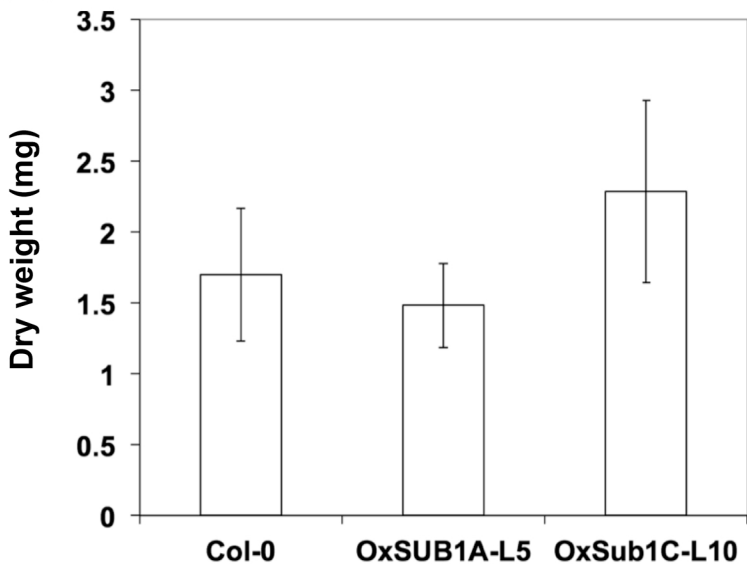

**(B)**

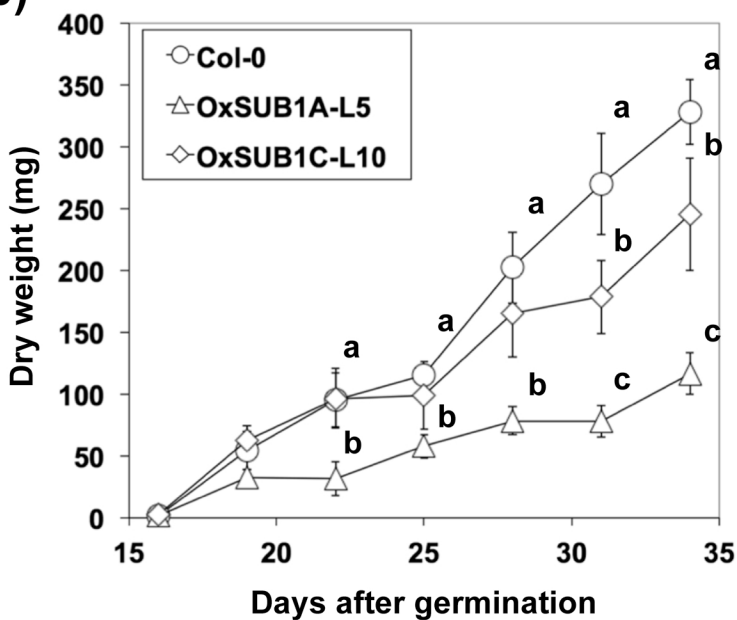

Supplement: Figure S3 — (A) Data of rosette tissue of 16 -day-old plants. (B) Data of above-ground tissue (rosette and cauline leaves, bolts and siliques). Different letters indicate significant difference with Col-0 on the same collection point (P < 0.05, Student’s t test). Values are means of n = 12 plants. Error bars are ±S.D. [file peerj-03-817-s003.pdf]

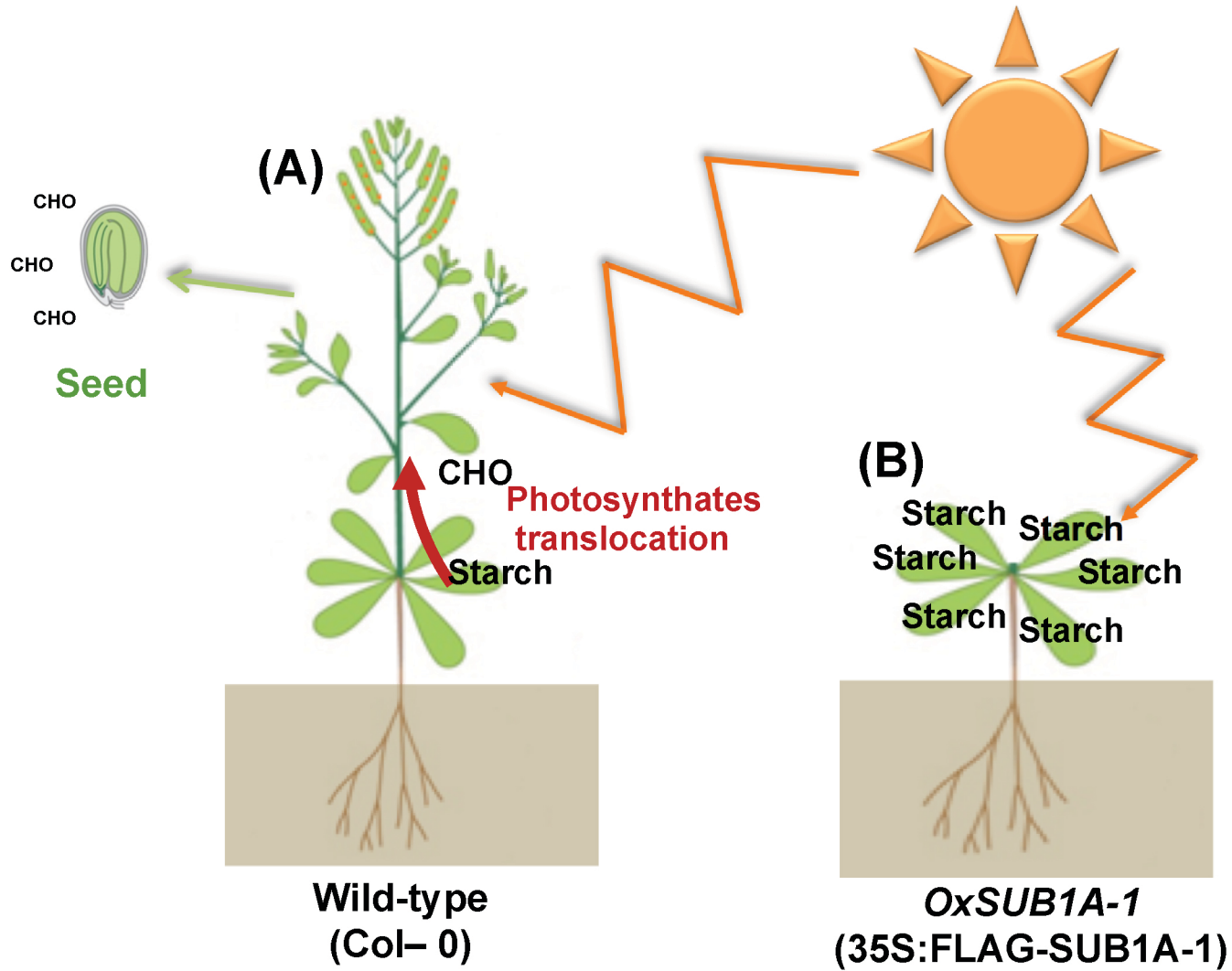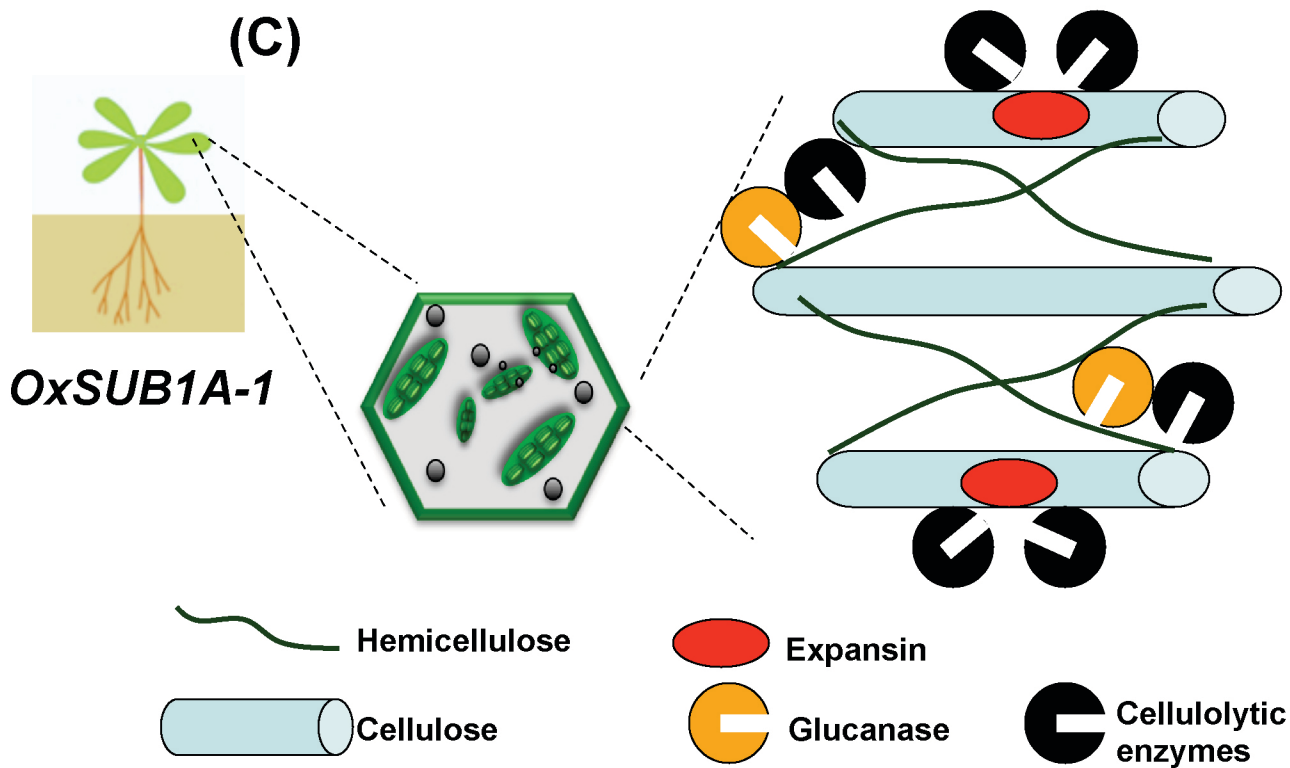

Supplement: Figure S4 — (A) Arabidopsis plants growing under normal conditions transit from juvenile to reproductive stage using the carbohydrates generated in photosynthesis as an energy resource for development of inflorescences and seeds. (B) OxSUB1A plants display flowering inhibition and starch conservation two characteristics of the rice LOQS response. The constitutive expression of these phenotypes under normal growth conditions leads to an improvement in amylolytic saccharification. (C) SUB1A-1 induces a set of cell wall associated proteins including expansin (AtEXP2) and glucan-1,3,-glucanase (BGL2) that act to weaken cell wall microfibrils and ease of access of external cellulolytic enzymes used for digestion of cellulose in biofuel production as compared to Col-0. CHO: soluble carbohydrates. [file peerj-03-817-s004.pdf]
