# Supplementary material for: Improvement of enzymatic saccharification yield in Arabidopsis thaliana by ectopic expression of the rice SUB1A-1 transcription factor
Source: PeerJ. 2015 Mar 3;3:e817. doi: 10.7717/peerj.817 (PMC4358655; doi:10.7717/peerj.817)
Supplement: Table S1 — Values are means of two independent experimental replicates ±S.D., each with n = 5 plants. [file peerj-03-817-s005.docx]

**Supplemental Table S1.** Polyphasic fluorescence rise (OJIP kinetics) of Col-0 and *Arabidopsis* 23-day-old plants ectopically expressing rice *SUB1A-1* and *SUB1C-1* genes. Values are means of two independent experimental replicates ±S.D., each with n=5 plants.

| **Genotype** | **Fv/Fm** | **Fm** | **Fo** | **Fv/Fo** |
| --- | --- | --- | --- | --- |
| Col-0 | 0.7921 ±0.0032 | 800.3 ±13.7 | 165.7 ±2.2 | 3.8315 ±0.07 |
| *OxSUB1A*-L5 | 0.8067 ±0.0026 | 771.8 ±19.7 | 148.8 ±4.0 | 4.1941 ±0.07 |
| *OxSUB1A*-L12 | 0.7865 ±0.0044 | 825.6 ±20.4 | 175.3 ±2.7 | 3.7087 ±0.08 |
| *OxSUB1C*-L6 | 0.7976 ±0.0023 | 894.8 ±24.2 | 180.6 ±3.9 | 3.9530 ±0.05 |
| *OxSUB1C*-L10 | 0.7840 ±0.0046 | 772.4 ±25.2 | 167.8 ±6.4 | 3.7232 ±0.09 |
