# Supplementary material for: Improvement of enzymatic saccharification yield in Arabidopsis thaliana by ectopic expression of the rice SUB1A-1 transcription factor
Source: PeerJ. 2015 Mar 3;3:e817. doi: 10.7717/peerj.817 (PMC4358655; doi:10.7717/peerj.817)
Supplement: Table S2 — Values are means of two independent experiments with three technical replicates each ±S.E, n = 25 seedlings. [file peerj-03-817-s006.docx]

**Supplemental Table S2.** Quantitative PCR of Col-0 and *Arabidopsis* 7-day-old seedlings ectopically expressing rice *SUB1A-1*. Values are means of two independent experiments with three technical replicates each ±S.E, n=25 seedlings.

| **Gene** | **Genotype** | **Ct ± S.E.** |
| --- | --- | --- |
| *ACT2*, At3G18780 | Col-0 | 27.28 ± 0.66 |
|  | *OxSUB1A*-L5 | 27.06 ± 0.23 |
|  | *OxSUB1A*-L12 | 27.50 ± 0.20 |
| *TUB2*, At1g65480 | Col-0 | 26.02 ± 0.15 |
|  | *OxSUB1A*-L5 | 25.4 ± 0.17 |
|  | *OxSUB1A*-L12 | 25.54 ± 0.08 |
| *GWD1*, At1g10760 | Col-0 | 25.88 ± 0.04 |
|  | *OxSUB1A*-L5 | 25.50 ± 0.29 |
|  | *OxSUB1A*-L12 | 26.37 ± 0.06 |
| *SEX4*, At3g52180 | Col-0 | 30.64 ± 0.51 |
|  | *OxSUB1A*-L5 | 30.72 ± 0.36 |
|  | *OxSUB1A*-L12 | 31.54 ± 0.35 |
| *SPL3*, At2g33810 | Col-0 | 24.46 ± 0.23 |
|  | *OxSUB1A*-L5 | 23.63 ± 0.10 |
|  | *OxSUB1A*-L12 | 24.88 ± 0.04 |
| *SPL4*, At1g53160 | Col-0 | 26.96 ± 0.20 |
|  | *OxSUB1A*-L5 | 26.94 ± 0.05 |
|  | *OxSUB1A*-L12 | 26.77 ± 0.16 |
| *SPL5*, At3g15270 | Col-0 | 28.33 ± 0.38 |
|  | *OxSUB1A*-L5 | 27.25 ± 0.02 |
|  | *OxSUB1A*-L12 | 27.63 ± 0.03 |
| *SOC1*, At2g45660 | Col-0 | 38.65 ± 0.43 |
|  | *OxSUB1A*-L5 | 37.12 ± 0.26 |
|  | *OxSUB1A*-L12 | 37.57 ± 0.28 |
| *FUL5*, At5g60910 | Col-0 | 31.99 ± 0.89 |
|  | *OxSUB1A*-L5 | 33.22 ± 0.1 |
|  | *OxSUB1A*-L12 | 32.85 ± 0.32 |
