# Supplementary material for: Improvement of enzymatic saccharification yield in Arabidopsis thaliana by ectopic expression of the rice SUB1A-1 transcription factor
Source: PeerJ. 2015 Mar 3;3:e817. doi: 10.7717/peerj.817 (PMC4358655; doi:10.7717/peerj.817)
Supplement: Table S3 — Grouped using GO and quantified by ATH1 microarray hybridization (Gene Expression Omnibus accession number GSE27669). [file peerj-03-817-s007.docx]

**Supplemental Table S3.** Expression values of cell wall genes significantly up regulated in 7-day-old complete seedlings of OxSUB1A-L5 when compared to Col-0 as grouped using GO and quantified by ATH1 microarray hybridization (Gene Expression Omnibus accession number GSE27669).

| **AGI** | **Affy ID** | **Description** | **logFC** | **adj.P.Val** |
| --- | --- | --- | --- | --- |
| AT3G57260 | 251625_at | BGL2 (PATHOGENESIS-RELATED PROTEIN 2); glucan 1,3-beta-glucosidase/ hydrolase, hydrolyzing O-glycosyl compounds | 4.568 | 6.85E-06 |
| AT1G21250 | 259561_at | WAK1 (CELL WALL-ASSOCIATED KINASE); kinase | 3.251 | 1.19E-05 |
| AT5G57550 | 247866_at | XTR3 (XYLOGLUCAN ENDOTRANSGLYCOSYLASE 3); hydrolase, acting on glycosyl bonds | 3.236 | 6.85E-06 |
| AT5G03350 | 250942_at | legume lectin family protein | 2.561 | 6.43E-06 |
| AT5G05290 | 250780_at | ATEXPA2 (ARABIDOPSIS THALIANA EXPANSIN A2) | 2.135 | 1.00E-03 |
| AT2G18660 | 266070_at | EXLB3 (EXPANSIN-LIKE B3 PRECURSOR) | 2.118 | 1.16E-03 |
| AT2G45220 | 245148_at | pectinesterase family protein | 1.553 | 1.08E-05 |
| AT1G47960 | 259616_at | C/VIF1 (CELL WALL / VACUOLAR INHIBITOR OF FRUCTOSIDASE 1); pectinesterase inhibitor | 1.529 | 1.14E-05 |
| AT2G02990 | 266743_at | RNS1 (RIBONUCLEASE 1); endoribonuclease | 1.514 | 6.85E-06 |
| AT5G05340 | 250798_at | peroxidase, putative | 1.401 | 3.63E-04 |
| AT1G32170 | 245794_at | XTR4 (XYLOGLUCAN ENDOTRANSGLYCOSYLASE 4); hydrolase, acting on glycosyl bonds | 1.396 | 1.24E-05 |
| AT1G02930;AT1G02920 | 262119_s_at | [AT1G02930, ATGSTF6 (EARLY RESPONSIVE TO DEHYDRATION 11); glutathione transferase];[AT1G02920, ATGSTF7 (GLUTATHIONE S-TRANSFERASE 11); glutathione transferase] | 1.357 | 5.98E-04 |
| AT4G19810 | 254543_at | glycosyl hydrolase family 18 protein | 1.315 | 2.60E-05 |
| AT5G22460 | 249917_at | esterase/lipase/thioesterase family protein | 1.146 | 2.30E-04 |
| AT3G13790 | 256787_at | ATBFRUCT1/ATCWINV1 (ARABIDOPSIS THALIANA CELL WALL INVERTASE 1); beta-fructofuranosidase/ hydrolase, hydrolyzing O-glycosyl compounds | 1.137 | 3.30E-05 |
| AT4G25810 | 254042_at | XTR6 (XYLOGLUCAN ENDOTRANSGLYCOSYLASE 6); hydrolase, acting on glycosyl bonds | 1.136 | 4.59E-04 |
| AT2G34930 | 267411_at | disease resistance family protein | 1.066 | 2.33E-04 |
| AT4G02330 | 255524_at | ATPMEPCRB; pectinesterase | 1.049 | 2.22E-02 |
